# Adverse Childhood Experiences and Maternal–Fetal Attachment: Indirect Associations via Prenatal Depressive Symptoms in a Romanian Sample

**DOI:** 10.3390/bs16060911

**Published:** 2026-06-03

**Authors:** Risvan Vlad Rusu, Dan Octavian Rusu, Cristian Delcea

**Affiliations:** 1 Department of Research, Institute of Sexology, 400394 Cluj-Napoca, Romania; office@sexology.ro; 2 Department of Applied Psychology, Babes-Bolyai University of Cluj-Napoca, 4305849 Cluj-Napoca, Romania; 3 Multidisciplinary Doctoral School, Vasile Goldiș Western University of Arad, 310025 Arad, Romania

**Keywords:** adverse childhood experiences, prenatal depressive symptoms, maternal–fetal attachment, perceived social support, perinatal mental health, pregnancy

## Abstract

Adverse childhood experiences (ACEs) have been associated with increased vulnerability to depressive symptoms during pregnancy and may also be related to emerging prenatal relational processes. This cross-sectional study examined whether prenatal depressive symptoms statistically accounted for the association between ACEs and maternal–fetal attachment (MFA), and whether this indirect association varied as a function of perceived social support from partners, family, and friends. The sample included 149 Romanian women in the first trimester of their first pregnancy. Participants completed self-report measures assessing ACEs, prenatal depressive symptoms, MFA, and perceived social support. Conditional process analyses were conducted using PROCESS Model 7, controlling for maternal age and perceived socioeconomic status. Higher ACEs were significantly associated with increased prenatal depressive symptoms (b = 0.43, *p* = 0.001), which in turn were associated with lower MFA (b = −0.03, *p* = 0.023). The indirect association between ACEs and MFA via prenatal depressive symptoms was statistically significant (b = −0.01, 95% CI [−0.028, −0.0009]). However, perceived social support from partners, family, and friends did not significantly moderate this indirect association. These findings provide preliminary evidence that prenatal depressive symptoms represent an important psychological correlate linking early-life adversity with lower MFA in early pregnancy. Given the cross-sectional design, findings should be interpreted as indirect associations rather than causal mediation.

## 1. Introduction

Adverse childhood experiences (ACEs), encompassing various forms of abuse, neglect, and household dysfunction occurring before the age of 18, represent a major developmental risk factor with long-term consequences for mental health and interpersonal functioning (Hughes et al., 2017; Osofsky et al., 2021; Wang et al., 2025). In the present study, ACEs are conceptualized as a cumulative index of early-life adversity. This approach is widely used in the literature and allows for the examination of dose–response associations between adversity and later outcomes (Hughes et al., 2017). However, cumulative ACE scores do not capture important qualitative aspects of adversity, such as severity, chronicity, or the specific type of experience. As such, they should be interpreted as indicators of overall exposure to adversity rather than precise representations of its underlying characteristics. A substantial and growing body of research has demonstrated that cumulative exposure to ACEs is robustly associated with elevated levels of depressive symptomatology across the lifespan, including during the perinatal period (Brown et al., 2021; Y. Zhang, 2025). These associations are typically interpreted within a developmental psychopathology framework, whereby early adversity disrupts emotional regulation, cognitive schemas, and stress reactivity, thereby increasing vulnerability to later affective disorders (Belsky & de Haan, 2011; Madigan et al., 2018).

Pregnancy represents a particularly sensitive developmental context in which maternal psychological functioning has implications not only for maternal well-being but also for emerging relational processes with the fetus (Rollè et al., 2020; Lefkovics et al., 2014). Among these processes, maternal–fetal attachment (MFA)—defined as the emotional bond that develops between a pregnant woman and her unborn child—has been identified as a key precursor of postnatal bonding and caregiving behaviors (Condon, 1993; Alhusen, 2008). Lower levels of MFA have been associated with poorer maternal adjustment, reduced caregiving sensitivity, and less optimal developmental outcomes in offspring (Alhusen, 2008; Rollè et al., 2020).

Importantly, the first trimester of pregnancy represents a period of heightened biological and psychological sensitivity, during which rapid hormonal changes and the psychological adjustment to pregnancy may increase vulnerability to depressive symptoms (Glynn et al., 2001; Lee et al., 2007). At the same time, early forms of MFA begin to emerge, making this stage particularly relevant for examining how maternal psychological functioning is associated with prenatal relational processes (Condon, 1993; Rollè et al., 2020). Although MFA is often conceptualized as becoming more pronounced in the later stages of pregnancy, emerging evidence suggests that early forms of prenatal attachment are detectable as early as the first trimester. During this period, cognitive and emotional representations of the fetus begin to develop, even in the absence of consistent physical cues such as fetal movement (Condon, 1993; Rollè et al., 2020; Yarcheski et al., 2009).

Examining MFA at this early stage may therefore provide valuable insight into the initial formation of prenatal relational processes, prior to their consolidation in later pregnancy. At the same time, attachment during the first trimester may be comparatively less stable, less differentiated, and less behaviorally expressed than in later stages of pregnancy, given the relative absence of consistent physical cues and the ongoing psychological adjustment to pregnancy. This has important implications for the interpretation of variability and effect sizes observed during early pregnancy (Condon, 1993; Rollè et al., 2020).

A consistent line of research, including both empirical studies and systematic reviews, has shown that prenatal depressive symptoms are negatively associated with MFA (Rollè et al., 2020; Goecke et al., 2012; L. Zhang et al., 2021; Berthelot et al., 2019). In parallel, evidence indicates that women with higher levels of early-life adversity are more likely to experience depressive symptoms during pregnancy (Brown et al., 2021; Swords et al., 2024). Taken together, these findings suggest a plausible pattern of associations in which early adversity is indirectly related to prenatal relational functioning via maternal depressive symptomatology.

Although prior research has begun to examine associations between adverse childhood experiences (ACEs), prenatal depressive symptoms, and maternal–fetal attachment (MFA), evidence remains limited and fragmented. Brown et al. (2021) provided one of the few empirical tests of an indirect association linking ACEs, prenatal depressive symptoms, and MFA within a single analytical framework. Importantly, findings were not consistently replicated across all cultural contexts included in the study, suggesting potential contextual variability rather than a uniformly established pattern. Moreover, findings regarding the direct association between ACEs and MFA remain mixed (Berthelot et al., 2019; Topal et al., 2024).

To date, no study, to our knowledge, has examined these associations specifically among women in the first trimester of pregnancy within a Romanian context while simultaneously considering different sources of perceived social support (i.e., partner, family, and friend support) within a moderated framework. Examining these associations during the first trimester may be particularly informative, as this stage represents an early and potentially less stable phase in the development of prenatal attachment during which depressive symptoms and psychological adjustment processes may be especially salient. Romania represents a particularly relevant context for examining these associations, given the relative scarcity of perinatal mental health research conducted in Eastern European populations compared to North American and Western European settings. Sociocultural differences in family structures, intergenerational support, attitudes toward pregnancy, and access to mental health services may shape how early adversity, depressive symptoms, and prenatal attachment are experienced and expressed during pregnancy. Examining these associations in a Romanian sample therefore contributes to the cross-cultural generalizability of existing findings.

Clarifying these associations is essential from both theoretical and applied perspectives. From a theoretical standpoint, identifying intermediary processes allows for a more precise understanding of how distal developmental risks relate to proximal relational outcomes, in line with intergenerational transmission models (Madigan et al., 2018; Belsky & de Haan, 2011). From an applied perspective, such knowledge can inform the development of targeted interventions during pregnancy, particularly by identifying modifiable psychological factors that may disrupt or support the development of prenatal attachment.

Accordingly, the present study aims to examine whether prenatal depressive symptoms account for the association between ACEs and MFA. In line with prior research and theoretical models, ACEs are conceptualized as a cumulative index of early-life adversity, prenatal depression as continuous depressive symptom severity, and MFA as a continuous indicator of emotional bonding toward the fetus. By testing this model, we seek to validate indirect associations between ACEs, prenatal depression and MFA.

In addition, we explore whether the indirect associations vary as a function of perceived social support, including support from partners, family, and friends. Prior literature suggests that different sources of social support may exert distinct influences on maternal psychological adjustment during pregnancy, with partner support often identified as particularly salient in relation to prenatal mental health and relational functioning.

Overall, this study contributes to the literature by advancing a more integrated understanding of how early-life adversity may be associated with prenatal relational processes through maternal depressive symptoms, while also evaluating the extent to which the associations vary as a function of perceived social support. Given the cross-sectional nature of the data, the present study focuses on indirect associations rather than causal mediation.

### Hypotheses

Building on the theoretical framework and empirical evidence outlined above, we propose the following four hypotheses.

First, consistent with extensive evidence linking early adversity to later affective vulnerability, higher levels of ACEs are expected to be associated with increased prenatal depressive symptoms. Prior research has consistently shown that exposure to early-life stress is associated with heightened levels of depressive symptomatology across the lifespan, including during pregnancy (Hughes et al., 2017; Brown et al., 2021; Swords et al., 2024). Therefore, we propose the following hypothesis:

**H1.**  *Higher levels of ACEs will be associated with higher levels of prenatal depressive symptoms.*

Second, given the well-documented association between prenatal depression and disruptions in emotional engagement during pregnancy, higher levels of depressive symptomatology are expected to be associated with lower levels of MFA. Depressive symptoms may interfere with emotional investment, reduce positive affect, and limit the development of internal representations of the fetus (Rollè et al., 2020). Thus, we put forward Hypothesis 2:

**H2.**  *Higher levels of prenatal depressive symptoms will be associated with lower levels of MFA.*

Third, through integrating these associations within a developmental framework, prenatal depressive symptoms are expected to account for the indirect association between early adversity and prenatal attachment. Developmental psychopathology models suggest that distal risk factors exert their influence through proximal psychological processes (Madigan et al., 2018; Belsky & de Haan, 2011). Given prior evidence linking ACEs to depression and depression to MFA, depressive symptomatology is expected to explain the indirect association between ACEs and MFA. Accordingly, Hypothesis 3 is proposed:

**H3.**  *Prenatal depressive symptoms will mediate the association between ACEs and MFA.*

Given prior theoretical and empirical work suggesting that social support may buffer the effects of stress and adversity on psychological functioning (Cohen & Wills, 1985; Swords et al., 2024), we explored whether the indirect association between ACEs and MFA via prenatal depressive symptoms may vary as a function of perceived social support from partners, family, and friends.

**H4.**  *The indirect association between ACEs and MFA via prenatal depressive symptoms may vary as a function of perceived social support, including support from partners, family, and friends, such that higher levels of perceived support may attenuate this association.*

## 2. Materials and Methods

### 2.1. Participants

The sample consisted of 149 pregnant women from Romania, with a mean age of 29.99 years (SD = 4.63; range = 18–43), all in the first trimester of their first pregnancy. Participants were recruited using a convenience sampling strategy through an online Romanian Facebook support group dedicated to first-time pregnant women. Participants resided in multiple regions of Romania, with the largest proportion reporting residence in Cluj-Napoca (39.6%), followed by Satu Mare (22.8%), Baia Mare (14.1%), Târgu Mureș (12.1%), and Bucharest (11.4%). The sample was predominantly composed of participants identifying as Romanian ethnicity (58.4%), followed by Hungarian (36.9%) and Roma (4.7%).

In terms of educational attainment, the sample was characterized by relatively high levels of education. Specifically, 37.2% held an undergraduate degree and 33.8% a master’s degree, while a smaller proportion reported doctoral-level education (2.7%). Lower levels of education were less frequent, with 13.5% having completed senior secondary/high school, 5.4% vocational or technical education, and 1.4% secondary school only. Additionally, 6.1% reported other forms of education. Overall, the sample was skewed toward higher educational attainment.

Inclusion criteria required participants to be at least 18 years old and currently pregnant in the first trimester at the time of data collection. Exclusion criteria included self-reported diagnosis of severe psychiatric disorders (e.g., psychotic disorders) and incomplete survey responses.

### 2.2. Procedure

Participants were recruited online using a convenience sampling strategy through a Romanian Facebook support group dedicated to first-time pregnant women. The group functions as a national-level online community focused on prenatal support, information sharing, and peer interaction among expectant mothers. The study was approved by the Ethics Committee of the Institute of Sexology, Cluj-Napoca, Romania (approval code: 517; date of approval: 12 November 2025). A study advertisement containing a brief description of the research aims and a link to the survey was posted in the group.

Participants accessed the questionnaire through a secure online platform (Google Forms). Prior to participation, all individuals were presented with an informed consent form describing the study aims, confidentiality procedures, voluntary nature of participation, and the right to withdraw at any time without penalty. Only participants who provided informed consent were allowed to proceed to the survey.

The survey was completed anonymously, and no personally identifiable information was collected. To minimize the likelihood of duplicate responses, the questionnaire was configured to allow a single submission per device and IP address. In addition, response patterns were screened for potential duplicates. Data were collected between November 2025 and March 2026, and no financial compensation was provided for participation.

All instruments were translated into Romanian using a forward–backward translation procedure conducted by independent bilingual translators in order to maximize linguistic and conceptual equivalence. Although some of these instruments or adapted versions have been used previously in Romanian or culturally similar populations, formal psychometric validation evidence remains limited for certain measures. Accordingly, internal consistency indices were examined in the present sample and indicated acceptable to good reliability across all scales.

### 2.3. Measures

#### 2.3.1. Adverse Childhood Experiences

ACEs were assessed using an adapted version of the World Health Organization Adverse Childhood Experiences International Questionnaire (ACE-IQ; WHO, 2018), as implemented within the Evidence for Better Lives Study (EBLS). The EBLS version includes 19 of the original 31 ACE-IQ items and assesses exposure to multiple forms of early adversity, including abuse, neglect, household dysfunction, and caregiver loss or separation. The EBLS version includes 19 of the original 31 ACE-IQ items and assesses exposure to multiple forms of early adversity, including abuse, neglect, household dysfunction, and caregiver loss or separation. The measure included dichotomous items reflecting exposure to various forms of early adversity, including abuse, neglect, and household dysfunction, as well as additional contextual stressors relevant to diverse sociocultural settings. Example items included “Did a parent or adult in your household often insult or humiliate you?” and “Did you live with anyone who was a problem drinker or alcoholic?”. Responses were coded as 0 (no) and 1 (yes), and summed to create a total ACE score, with higher values indicating greater exposure to adversity. In the present study, internal consistency was good (α = 0.78).

#### 2.3.2. Prenatal Depressive Symptoms

Prenatal depressive symptoms were measured using the Patient Health Questionnaire-9 (PHQ-9; Kroenke et al., 2001). This instrument consists of nine items assessing the frequency of depressive symptoms over the past two weeks. Responses are rated on a 4-point Likert scale ranging from 0 (not at all) to 3 (nearly every day), yielding a total score ranging from 0 to 27.

The EBLS adaptation and cross-cultural psychometric evaluation of the PHQ-9 across multiple EBLS sites has been previously described by Murray et al. (2022), including translation procedures, reliability analyses, and cross-country measurement invariance testing.

In the present study, depressive symptoms were operationalized as a continuous variable, with total scores computed by summing the items, such that higher scores indicate greater symptom severity. Although established cut-off scores are commonly used to indicate clinically relevant levels of depression (e.g., scores ≥10 reflecting moderate depressive symptoms), these thresholds were not applied in the current analyses.

The PHQ-9 does not include subscales, and all items contribute to a single total score. Internal consistency in the current sample was acceptable (α = 0.77).

#### 2.3.3. Maternal–Fetal Attachment

Maternal–fetal attachment (MFA) was assessed using the 18-item Prenatal Attachment Inventory-Revised (PAI-R; adapted from Muller, 1993), as implemented within the Evidence for Better Lives Study (EBLS). Previous EBLS work supported the cross-cultural applicability of the measure across diverse low- and middle-income settings. The scale assesses emotional bonding and engagement with the fetus during pregnancy, including maternal thoughts, feelings, and behaviors directed toward the unborn child. Example items included “I stroke my tummy to communicate with the baby” and “I tell others about the baby”. Responses were rated on a 4-point Likert scale ranging from 1 (almost never) to 4 (almost always), with higher scores indicating stronger prenatal attachment. Item scores were averaged to obtain a mean MFA score, with higher values reflecting stronger maternal–fetal attachment.

In the present sample, internal consistency was acceptable (α = 0.75).

#### 2.3.4. Perceived Social Support

Perceived social support from family and friends was assessed using the corresponding subscales of the Multidimensional Scale of Perceived Social Support (MSPSS; Zimet et al., 1988). Items are rated on a 5-point Likert scale ranging from 1 (strongly disagree) to 5 (strongly agree), with higher scores indicating greater perceived support.

Perceived partner support was assessed using the Partner Supportiveness Scale implemented within the Evidence for Better Lives Study (EBLS; Valdebenito et al., 2020). This measure includes five items assessing key dimensions of partner supportiveness within the relationship, including compromise, affection, help, listening, and understanding. Items were rated on a 5-point Likert scale ranging from 1 (never) to 5 (always), with higher scores indicating greater perceived partner support.

For all social support measures, item scores were averaged to obtain mean subscale scores. Family support, friend support, and partner support were analysed separately, and no global social support score was computed.

#### 2.3.5. Demographic Factors

Covariates were selected based on the literature linking maternal age and socioeconomic conditions to both antenatal depressive symptoms and MFA. Although demographic factors such as age and socioeconomic indicators tend to show relatively small associations with MFA, meta-analytic evidence suggests that they represent consistent background correlates of prenatal bonding (Yarcheski et al., 2009). In addition, socioeconomic disadvantage has been robustly associated with increased risk for perinatal depressive symptoms (Biaggi et al., 2016; Gavin et al., 2005).

Given the modest sample size and the complexity of the moderated indirect models, a parsimonious covariate strategy was adopted to control for broad demographic influences while minimizing the risk of model overfitting.

Thus, two covariates were included in all analyses. Maternal age was measured in years as a continuous variable. Perceived socioeconomic status was assessed using the MacArthur Scale of Subjective Social Status (Adler et al., 2000), a single-item measure in which participants indicate their perceived position in society on a visual 10-rung ladder, with higher rungs representing higher perceived socioeconomic status.

### 2.4. Statistical Analysis

Analyses were conducted using SPSS version 30 and the PROCESS macro (version 5.0v; Hayes, 2022). Conditional process analysis combines mediation and moderation approaches within a single analytical framework, allowing the examination of whether an indirect association varies across levels of a moderator variable (Hayes, 2022). Model 7 was used to test conditional indirect associations, with ACEs as the independent variable, prenatal depressive symptoms as the mediator, MFA as the outcome, and perceived social support as the moderator.

To examine the potential influence of different relational contexts, three separate models were tested using perceived support from partners, family, and friends as moderators. Maternal age and perceived socioeconomic status were included as covariates in all models.

Continuous predictors were mean-centered prior to analysis. Indirect effects were estimated using bias-corrected bootstrap confidence intervals based on 5000 resamples. An indirect effect was considered statistically significant if the 95% confidence interval did not include zero.

Given the sample size, a sensitivity analysis was conducted using G*Power 3.1 to estimate the smallest detectable effect size with 80% power and α = 0.05. The results indicated that the present study was adequately powered to detect small-to-moderate main effects, but may have been underpowered for detecting smaller interaction effects. Accordingly, null findings regarding moderation should be interpreted cautiously.

Because three separate moderation models were tested, findings regarding interaction and moderated indirect effects were interpreted cautiously in light of the increased number of statistical tests conducted.

There were no missing data because all survey items were configured as mandatory within the online questionnaire platform. As a result, incomplete survey submissions were not possible.

## 3. Results

The descriptive statistics and bivariate correlations among the study variables are presented in Table 1.

To test the proposed hypotheses, conditional process analyses were conducted using the PROCESS macro for SPSS (Model 7; Hayes, 2022) to examine the indirect association between ACEs and MFA via the mediation of prenatal depressive symptoms, and to assess whether this indirect effect varied as a function of perceived social support (partner, family, and friend support). Maternal age and perceived socioeconomic status were included as covariates. Given the cross-sectional design, the results are interpreted in terms of indirect associations rather than causal mediation. Regression coefficients for the conditional process models are presented in Table 2.

In line with Hypothesis 1, higher levels of ACEs were significantly associated with increased prenatal depressive symptoms, *b* = 0.43, *SE* = 0.13, *t* (143) = 3.37, *p* = 0.001. Consistent with Hypothesis 2, prenatal depressive symptoms were significantly negatively associated with MFA, *b* = −0.03, *SE* = 0.01, *t* (144) = −2.29, *p* = 0.023, indicating that higher depressive symptom severity was related to lower levels of attachment.

Addressing Hypothesis 3, the indirect association between ACEs and MFA through prenatal depressive symptoms was statistically significant, as indicated by bootstrap confidence intervals that did not include zero (*b* = −0.01, 95% CI [−0.028, −0.0009]). Specifically, higher levels of early adversity were associated with lower MFA indirectly through increased prenatal depressive symptomatology.

Regarding Hypotheses 4a–4c, the interaction terms between ACEs and perceived social support (partner, family, and friend support) in predicting prenatal depressive symptoms were not significant across all models (partner: *b* = 0.22, *SE* = 0.32, *t*(143) = 0.68, *p* = 0.500; family: *b* = 0.19, *SE* = 0.22, *t*(143) = 0.87, *p* = 0.387; friends: *b* = −0.09, *SE* = 0.18, *t*(143) = −0.50, *p* = 0.621).

Conditional indirect effects were examined at low, mean, and high levels of each moderator. Although some conditional indirect effects reached statistical significance at specific levels of perceived support, this pattern should be interpreted cautiously. Importantly, the indices of moderated indirect effects were not significant for any of the moderators (partner: index = −0.01, BootSE = 0.01, 95% CI [−0.030, 0.010]; family: index = −0.01, BootSE = 0.01, 95% CI [−0.024, 0.004]; friends: index = 0.003, BootSE = 0.005, 95% CI [−0.006, 0.016]). These findings indicate that there was no reliable evidence that the indirect association linking ACEs to MFA via prenatal depressive symptoms varied as a function of perceived social support.

For descriptive completeness, the conditional indirect effects are reported as follows. For perceived partner support, the indirect effect remained statistically significant at low (b = −0.01, 95% CI [−0.025, −0.0003]), mean (b = −0.01, 95% CI [−0.028, −0.0009]), and high (b = −0.02, 95% CI [−0.038, −0.0008]) levels. For perceived family support, the indirect effect was not significant at low levels (b = −0.01, 95% CI [−0.025, 0.002]) but was significant at mean (b = −0.02, 95% CI [−0.035, −0.001]) and high (b = −0.02, 95% CI [−0.035, −0.001]) levels. For perceived friend support, the indirect effect was significant at low (b = −0.01, 95% CI [−0.032, −0.001]) and mean (b = −0.01, 95% CI [−0.028, −0.001]) levels, but not at high levels (b = −0.01, 95% CI [−0.028, 0.0001]).

## 4. Discussion

The present study examined the indirect association between ACEs and MFA through prenatal depressive symptoms, as well as the potential moderating role of perceived social support. Overall, the findings support a coherent pattern in which early-life adversity is associated with prenatal relational functioning primarily through maternal depressive symptomatology, with this pattern of associations appearing consistent across different levels of perceived social support.

### 4.1. Hypothesis 1: ACEs→Prenatal Depression

Consistent with Hypothesis 1, higher levels of adverse childhood experiences were significantly associated with increased prenatal depressive symptoms. This finding aligns with a robust body of literature demonstrating that early-life adversity constitutes a major risk factor for depression across the lifespan (Chapman et al., 2004; Hughes et al., 2017). From a developmental psychopathology perspective, ACEs are thought to disrupt emotion regulation systems, stress responsivity, and cognitive schemas, thereby increasing vulnerability to affective disorders (McLaughlin et al., 2010; Madigan et al., 2018).

In the context of pregnancy, this vulnerability may be particularly pronounced. Pregnancy represents a period of heightened psychological sensitivity, during which unresolved trauma may resurface and interact with current stressors (Seng et al., 2001). Previous studies have shown that women with a history of childhood adversity report higher levels of prenatal depressive symptoms and emotional distress (Gavin et al., 2005; Biaggi et al., 2016), supporting the present findings. Taken together, these results reinforce the notion that ACEs exert a long-term influence on mental health, extending into critical developmental periods such as pregnancy.

### 4.2. Hypothesis 2: Prenatal Depression→Maternal–Fetal Attachment

In line with Hypothesis 2, prenatal depressive symptoms were negatively associated with MFA. This finding is consistent with prior research indicating that depression during pregnancy can interfere with emotional bonding processes (Alhusen et al., 2012; Yarcheski et al., 2009).

From a theoretical standpoint, depressive symptoms such as anhedonia, emotional withdrawal, and reduced cognitive engagement may limit a mother’s ability to form a meaningful psychological connection with the fetus (Lindgren, 2001). Moreover, depression has been associated with diminished responsiveness to emotional stimuli and reduced mentalization capacities, both of which are essential for the development of prenatal attachment (Joormann & Gotlib, 2006; Sharp et al., 2010). Importantly, the present findings extend this body of literature by demonstrating that even in a relatively well-functioning community sample, variations in depressive symptom severity are meaningfully associated with differences in MFA.

### 4.3. Hypothesis 3: Indirect Association (ACEs→Depression→Attachment)

Addressing Hypothesis 3, the results indicated a significant indirect association between ACEs and MFA through prenatal depressive symptoms. This finding suggests that early adversity may be associated with lower prenatal bonding indirectly by increasing vulnerability to depressive symptomatology. At the same time, the observed indirect effect was relatively small, and the bivariate association between ACEs and MFA was near zero. This pattern suggests that early adversity may not be directly associated with prenatal attachment in a robust or uniform manner, but rather through more proximal psychological processes such as depressive symptomatology.

This pattern is consistent with cumulative risk and developmental cascade models, which posit that distal risk factors exert their influence through proximal psychological processes (Evans et al., 2013; Madigan et al., 2018). In this context, depressive symptoms appear to represent a key statistical intermediary association in the link between early adversity and current relational functioning.

Although relatively few studies have examined this exact pathway in the prenatal period, existing evidence supports similar patterns of associations. For example, childhood trauma has been associated with impairments in maternal representations and bonding, partially explained by maternal psychopathology (Lyons-Ruth & Block, 1996; Muzik et al., 2013). The present study extends these findings by situating these patterns of associations specifically within the prenatal context, thereby contributing to a more integrated understanding of how early adversity may shape emerging maternal–infant relationships even before birth.

### 4.4. Hypotheses 4a–4c: Moderated Indirect Effects (Role of Social Support)

Contrary to Hypotheses 4a–4c, the indirect association between ACEs and maternal–fetal attachment through prenatal depressive symptoms did not significantly vary as a function of perceived support from partners, family, or friends. In addition, the interaction effects between ACEs and each form of perceived support in predicting depressive symptoms were not significant.

At first glance, these findings may appear inconsistent with the buffering hypothesis, which suggests that social support mitigates the negative effects of stress and adversity (Cohen & Wills, 1985). Indeed, prior studies have demonstrated that perceived support—particularly from intimate partners—can reduce prenatal depressive symptoms and improve maternal adjustment (Leahy-Warren et al., 2012; Stapleton et al., 2012). However, the present findings do not provide empirical support for a buffering effect of perceived social support within the tested model (Alhusen et al., 2013).

Several explanations may account for this pattern. First, social support may exert primarily direct effects on well-being rather than moderating effects, as suggested by prior theoretical and empirical work (Lakey & Orehek, 2011). Second, the relatively high levels of perceived support in the present sample may have restricted variability, thereby reducing statistical power to detect interaction effects. Third, the impact of early adversity may be sufficiently robust such that it is not easily attenuated by current relational resources, particularly within cross-sectional designs.

Importantly, the non-significant indices of moderated indirect effects across all models indicate that the indirect pathway linking ACEs to MFA via depressive symptoms appeared statistically similar across levels of perceived social support in this sample.

This suggests that, within this sample, social support does not significantly alter the underlying patterns of associations through which early adversity is associated with prenatal bonding.

### 4.5. Limitations

Several limitations should be considered when interpreting these findings. First, the cross-sectional design precludes causal inference, particularly regarding the temporal ordering of the observed associations. Although the proposed model is theoretically grounded, longitudinal data would be necessary to establish temporal precedence.

The sample size (N = 149) is adequate for detecting small-to-moderate main effects; however, statistical power may have been more limited for detecting interaction effects, which typically require larger samples. As such, the non-significant moderation findings should be interpreted cautiously, as smaller interaction effects may not have been detected (i.e., potential Type II error). In addition, the sample size may have limited the ability to detect smaller differences between specific sources of social support (e.g., partner, family, and friend support). Although statistically significant, the observed indirect effect sizes were relatively small and should therefore be interpreted with appropriate caution regarding their clinical magnitude. Second, all variables were assessed using self-report measures, raising the possibility of shared method variance and reporting biases. This limitation is particularly salient for the assessment of ACEs, which relied on retrospective reporting and may be subject to recall bias, including memory distortions and inaccuracies in the reporting of early-life adversity. Consequently, the observed associations may partially reflect biases in the retrospective reporting of both past experiences and current psychological states. Third, the sample was characterized by relatively high levels of perceived social support and educational attainment. While this reflects the specific recruitment context, it may limit the generalizability of the findings to more socioeconomically disadvantaged or clinically at-risk populations. Future research would benefit from examining these associations in more diverse samples, including individuals with varying levels of social support and socioeconomic resources. In addition, the convenience sampling recruitment strategy (online via social media) may have introduced selection bias, as reflected in the relatively high levels of education and perceived social support in the sample. This approach may underrepresent individuals from more socioeconomically disadvantaged backgrounds, those with limited access to online platforms, or those less engaged in prenatal support networks. As a result, the sample may not fully reflect the broader population of pregnant women.

The use of global measures of social support may have obscured more nuanced effects, such as differences between emotional versus instrumental support or perceived versus received support. Furthermore, the cumulative ACE measure, based on dichotomous items, does not capture the severity, frequency, or chronicity of early adverse experiences, which may limit the precision of the construct.

### 4.6. Implications and Contributions

Despite these limitations, the present study makes several important contributions. First, it provides a conceptual replication of a previously reported pattern of indirect associations linking ACEs to MFA via prenatal depressive symptoms. Specifically, Brown et al. (2021) identified this pattern in the third trimester of pregnancy, where prenatal depressive symptoms fully mediated the association in a large multi-country sample. The present study extends these findings by demonstrating a similar pattern of indirect associations at an earlier stage of pregnancy and within a Romanian sample, thereby extending existing evidence beyond predominantly North American and Western European populations and offering important cross-cultural support for the generalizability of this pattern from the early stages of pregnancy (Alhusen et al., 2013).

Second, it provides empirical support for a theoretically meaningful pattern of associations linking early adversity to prenatal relational functioning through depressive symptoms (Madigan et al., 2018; Muzik et al., 2013). It highlights prenatal depressive symptomatology as a key factor associated with variations in MFA (Alhusen et al., 2012; Yarcheski et al., 2009).

From a clinical perspective, these findings suggest that screening for childhood adversity and depressive symptoms during pregnancy may help identify women at risk of difficulties in prenatal attachment (Gavin et al., 2005; Biaggi et al., 2016). Interventions targeting maternal mental health may, in turn, support the development of prenatal bonding and potentially contribute to more adaptive postnatal caregiving processes (Stein et al., 2014; Letourneau et al., 2017).

### 4.7. Future Directions

Future research should employ longitudinal designs to examine the temporal dynamics of these associations and to test whether changes in depressive symptoms are associated with subsequent changes in MFA. Future studies would also benefit from employing more diverse and inclusive recruitment strategies to ensure broader representation across socioeconomic and educational backgrounds. In addition to online recruitment, approaches such as collaboration with healthcare providers, community-based organizations, and prenatal clinics may facilitate the inclusion of more socioeconomically disadvantaged or less digitally connected populations, thereby improving the generalizability of findings. Additionally, more refined assessments of social support may help clarify whether specific types of support (e.g., emotional, instrumental, or informational) and sources (e.g., partner, family, or peers) exert differential buffering effects under certain conditions, rather than treating social support as a global construct. Future research may also benefit from examining whether specific forms of childhood adversity exert differential associations with prenatal depressive symptoms and maternal–fetal attachment, rather than relying exclusively on cumulative ACE indices.

It would also be valuable for future research to examine alternative moderating factors using clearly specified and validated measures. For example, resilience could be assessed using instruments such as the Connor–Davidson Resilience Scale (CD-RISC), and emotion regulation capacities using measures such as the Emotion Regulation Questionnaire (ERQ). These variables could then be incorporated into moderated mediation or longitudinal models to test whether they buffer or amplify the association between adverse childhood experiences and prenatal outcomes over time. In addition, trauma-specific processes, such as trauma-related cognitions or symptoms, could be assessed using standardized self-report measures to further clarify underlying mechanisms. Beyond individual-level factors, future studies could adopt multilevel designs by combining individual survey data with contextual indicators (e.g., neighborhood socioeconomic status, access to healthcare services, or community-level social support) obtained from administrative or geocoded data sources. Such approaches would allow for the simultaneous examination of individual and contextual influences on maternal psychological functioning during pregnancy and provide a more comprehensive understanding of how early-life adversity is associated with prenatal relational processes across ecological levels.

## 5. Conclusions

The present study provides evidence for a coherent pattern in which ACEs are indirectly associated with MFA through prenatal depressive symptoms. Specifically, higher levels of early-life adversity were linked to increased depressive symptomatology during pregnancy, which in turn was associated with lower levels of prenatal attachment. These findings highlight prenatal depression as a key psychological process through which distal developmental risk may be related to emerging maternal–fetal relational functioning.

Contrary to expectations, perceived social support from partners, family, and friends did not significantly moderate this indirect association. This suggests that, within this sample, the pathway linking early adversity to prenatal attachment via depressive symptoms operates relatively consistently across different levels of perceived support.

Future longitudinal research is needed to clarify the temporal dynamics of these associations, particularly to determine whether changes in prenatal depressive symptoms precede subsequent changes in maternal–fetal attachment, and to better disentangle the directionality and potential bidirectional influences among early adversity, maternal mental health, and prenatal relational functioning.

## Figures and Tables

**Table 1:** Descriptive statistics and correlations among study variables (N = 149).

Variable	M	SD	1	2	3	4	5	6	7	8
1. ACEs	2.89	2.25	-							
2. PHQ-9	5.13	3.49	0.28 **	-						
3. MFA	3.16	0.54	−0.01	−0.17 *	-					
4. Partner support	4.58	0.44	−0.20 *	−0.14	0.15	-				
5. Family support	4.63	0.60	−0.24 **	−0.02	0.04	0.30 **	-			
6. Friend support	4.35	0.66	−0.14	−0.05	0.17 *	0.32 **	0.59 **	-		
7. Age	29.99	4.63	0.09	−0.04	−0.16	−0.14	−0.02	0.01	-	
8. Perceived social status	6.99	1.22	−0.19 *	−0.04	−0.00	0.15	0.16	0.12	0.10	-

Note: M—mean; SD—standard deviation; ACEs—adverse childhood experiences; PHQ-9—prenatal depressive symptoms; MFA—maternal–fetal attachment. * *p* < 0.05, ** *p* < 0.01.

**Table 2:** Regression coefficients for the conditional process models predicting prenatal depressive symptoms and maternal–fetal attachment.

Predictor	*b*	*SE*	*t*	*p*	95% CI
**Model 1: Partner Support as Moderator**					
ACEs→Prenatal depressive symptoms	0.432	0.129	3.37	0.001	[0.179, 0.686]
Partner support	−0.878	0.661	−1.33	0.186	[−2.179, 0.424]
ACEs × Partner support	0.216	0.321	0.68	0.500	[−0.417, 0.850]
Maternal age	−0.056	0.060	−0.94	0.351	[−0.175, 0.062]
Perceived social status	0.103	0.205	0.50	0.615	[−0.301, 0.507]
Prenatal depressive symptoms→MFA	−0.034	0.015	−2.29	0.023	[−0.063, −0.005]
**Model 2: Family Support as Moderator**					
ACEs→Prenatal depressive symptoms	0.430	0.128	3.36	0.001	[0.177, 0.683]
Family support	−0.490	0.445	−1.10	0.275	[−1.370, 0.389]
ACEs × Family support	0.194	0.223	0.87	0.387	[−0.247, 0.634]
Maternal age	−0.055	0.060	−0.92	0.360	[−0.174, 0.063]
Perceived social status	0.101	0.204	0.49	0.621	[−0.302, 0.504]
Prenatal depressive symptoms→MFA	−0.034	0.015	−2.29	0.023	[−0.063, −0.005]
**Model 3: Friend Support as Moderator**					
ACEs→Prenatal depressive symptoms	0.431	0.128	3.36	0.001	[0.178, 0.684]
Friend support	−0.182	0.378	−0.48	0.633	[−0.929, 0.564]
ACEs × Friend support	−0.091	0.181	−0.50	0.621	[−0.448, 0.266]
Maternal age	−0.056	0.060	−0.93	0.353	[−0.175, 0.062]
Perceived social status	0.102	0.205	0.50	0.618	[−0.302, 0.506]
Prenatal depressive symptoms→MFA	−0.034	0.015	−2.29	0.023	[−0.063, −0.005]

Note. ACEs = adverse childhood experiences; MFA = maternal–fetal attachment. Unstandardized regression coefficients are reported. Confidence intervals are bias-corrected bootstrap intervals based on 5000 resamples. All models controlled for maternal age and perceived socioeconomic status.

## Data Availability

The data presented in this study are available on request from the corresponding author. The data are not publicly available due to ethical restrictions.
